# Single Directional SMO Algorithm for Least Squares Support Vector Machines

**DOI:** 10.1155/2013/968438

**Published:** 2013-02-18

**Authors:** Xigao Shao, Kun Wu, Bifeng Liao

**Affiliations:** ^1^School of Mathematics and Statistics, Central South University, Changsha, Hunan 41007, China; ^2^Wengjing College, Yantai University, Yantai, Shandong 264005, China; ^3^School of Mathematics and Information Science, Yantai University, Yantai, Shandong 264005, China

## Abstract

Working set selection is a major step in decomposition methods for training least squares support vector machines (LS-SVMs). In this paper, a new technique for the selection of working set in sequential minimal optimization- (SMO-) type decomposition methods is proposed. By the new method, we can select a single direction to achieve the convergence of the optimality condition. A simple asymptotic convergence proof for the new algorithm is given. Experimental comparisons demonstrate that the classification accuracy of the new method is not largely different from the existing methods, but the training speed is faster than existing ones.

## 1. Introduction

In a classification problem, we consider a set of training samples, that is, the input vectors {**x**_*k*_}_*k*=1_^*N*^ along with corresponding class labels {*y*_*k*_}_*k*=1_^*N*^. Our task is to find a deterministic function that best represents the relation between input vectors and class labels. For classification or forecasting problems in machine learning, support vector machine (SVM) has been adopted in many applications because of its high precision [1–4]. SVMs require the solution of a quadratic programming problem. Another successful method for machine learning is least squares support vector machine (LS-SVM) [5]. Instead of solving a quadratic programming problem as in SVMs, the solutions of a set of linear equations are obtained in LS-SVMS. There are many proposed algorithms for training LS-SVMs: Suykens et al. proposed an iterative algorithm based on conjugate gradient (CG) algorithms [6]; Ferreira et al. presented a gradient system which can train the LS-SVM model [7] effectively; Chua introduced efficient computations for large least square support vector machine classifiers [8]; Chu et al. improved the efficiency of the CG algorithm by using one reduced system of linear equations [9]; Keerthi and Shevade extended the sequential minimal optimization (SMO) algorithms to solve the linear equations in LS-SVMs where the maximum violating pair (MVP) was selected as the working set [10]; based on the idea of SMO algorithm, Lifeng Bo et al. presented an improved method for working set selection by using functional gain (FG) [11]; Jian et al. designed a multiple kernel learning algorithm for LS-SVMs by convex programming [12]; and so on. These numerical algorithms are computationally attractive. Empirical comparisons show that SMO algorithm is more efficient than CG one for the large scale datasets.

Fast SVM training speed with SMO algorithm is an important goal for practitioners and many other proposals have been given for this in the literature. Initially, Platt presented two heuristics that resulted in a bit cumbersome selection [13]. Later, Keerthi et al. introduced the concept of a violating pair to denote two coefficients which cause a violation in the KKT optimality conditions of the dual, and the authors suggested to select always the pair that violated them the most, that is, the maximum violating pair (MVP) [14]. Finally, Fan et al. proposed a second order selection that usually results in faster training than the MVP rule [15]. By the above improvement, we can decrease the computational expense of SMO algorithm, while there are repeated selections of some concrete updating patterns in sequential minimal optimization. They are called training cycles. Barbero et al. studied the presence of them from a geometrical point of view [16]. They pointed out that the training cycles can be partially collapsed in a single updating vector that gave better optimal directions. The idea for training cycles can reduce the number of iterations and kernel operations for SMO algorithm.

Inspired by Barbero et al. [16], we present a single directional SMO algorithm for LS-SVMs, abbreviated as SD-SMO algorithm. In optimization procedure, an adaptive objective function is selected, and the single directional steps are given for the lagrangian multipliers, which can lessen the number of training cycles and further reduce iterations and kernel operations for SMO algorithm. Experiments show that the training time for LS-SVMs by SD-SMO algorithm can be reduced significantly, and it has a testing accuracy which is not largely different from traditional SMO algorithm.

The rest of this paper has the following structure. In the next section, LS-SVMs are briefly reviewed. In Section 3, SD-SMO algorithm for LS-SVMs is provided and the convergence of the improved algorithm is proved theoretically. Based on standard datasets, computational experiments describing the effectiveness of the improved algorithm are presented in Section 4. Finally, Section 5 is devoted to concluding remarks.

## 2. LS-SVM

In this section, we concisely review the basic principles of LS-SVMs. Given a training dataset of *N* points {**x**_*k*_, *y*_*k*_}_*k*=1_^*N*^ with input data **x**_*k*_ ∈ *R*^*n*^ and output data *y*_*k*_ ∈ *R*, we consider the following optimization problem in primal weight space:

(1) $$\begin{array}{c} \underset{w,b,e}{\min}\;J\left(w,e\right)=\frac{1}{2}w^{T}w+\frac{1}{2}\gamma \sum_{k=1}^{N}e_{k}^{2}, \end{array}$$

such that

(2) $$\begin{array}{c} y_{k}-\left(w^{T}\varphi \left(x_{k}\right)+b\right)=e_{k},\;k=1,2,\ldots ,N, \end{array}$$

where *γ* is a regularization factor, *e*_*k*_ is the difference between the desired output *y*_*k*_ and the actual output, and *φ*(·) is a nonlinear function mapping the data points into a high-dimensional Hibert space; in addition, the dot product in the high-dimensional space is equivalent to a positive-definite kernel function **k**(**x**_*i*_, **x**_*j*_) = *φ*(**x**_*i*_)^*T*^ *φ*(**x**_*j*_).

In primal weight space, a linear classifier in the new space takes the following form:

(3) $$\begin{array}{c} y\left(x\right)=\mathrm{sign}\left(w\cdot \varphi \left(x\right)+b\right). \end{array}$$

The weight vector *w* may be infinite dimensional; hence, using (1) to find the solutions is impossible in general. In order to solve this problem, we would compute the model in the dual space instead of the primal space. Let *b* = 0, and the simple problem without a bias term is considered in this paper as in the paper by Keerthi and Shevade [10]. The Lagrangian for the simple problem is

(4) $$\begin{array}{c} L\left(w,e;\alpha \right)=J\left(w,e\right)-\sum_{k=1}^{N}\alpha_{k}\left\{w^{T}\varphi \left(x_{k}\right)+e_{k}-y_{k}\right\}, \end{array}$$

where *α*_*i*_ are Lagrangian multipliers and are called support values. The Karush-Kuhn-Tucker (KKT) conditions for optimality are

(5) $$\begin{array}{c} \frac{\partial L}{\partial w}=0\to w=\sum_{k=1}^{k=N}\alpha_{k}y_{k}\varphi \left(x_{k}\right),\\ \frac{\partial L}{\partial e_{k}}=0\to \alpha_{k}=\gamma e_{k},\;k=1,\ldots ,N,\\ \frac{\partial L}{\partial \alpha_{k}}=0\to w^{T}\varphi \left(x_{k}\right)+e_{k}-y_{k}=0,\;k=1,\ldots ,N. \end{array}$$

After elimination of *w* and *e*, we could obtain the following linear system:

(6) $$\begin{array}{c} \left(K+\frac{I}{\gamma }\right)\alpha =y, \end{array}$$

where *y* = [*y*_1_, *y*_2_,…, *y*_*N*_]^*T*^, *α* = [*α*_1_, *α*_2_,…*α*_*N*_]^*T*^, and *K* ∈ *R* ^*N*×*N*^ is the kernel matrix. By solving the linear system (6), *α*_*i*_′*s* are obtained; hence, LS-SVM greatly simplifies the problem. The resulting LS-SVM model for function estimation is

(7) $$\begin{array}{c} y\left(x\right)=\sum_{k=1}^{N}\alpha_{k}k\left(x,x_{k}\right). \end{array}$$

For the choice of the kernel function **k**(·, ·), there are several possibilities: **k**(**x**, **x**_*k*_) = **x**_*k*_^*T*^ **x** (linear LS-SVM); **k**(**x**, **x**_*k*_) = (**x**_*k*_^*T*^ **x** + 1)^*d*^ (polynomial LS-SVM of degree *d*); **k**(**x**, **x**_*k*_) = exp⁡{−||**x**−**x**_*k*_||_2_^2^/*σ* ^2^} (RBF LS-SVM); **k**(**x**, **x**_*k*_) = tanh(*k* **x**_*k*_^*T*^ **x** + *θ*) (MLP LS-SVM). In this case, we focus on the choice of an RBF LS-SVM for the sequel. When solving large linear systems, we should apply iterative methods to (6), which was introduced by Jiao et al. [17]. The speed of convergence depends on the condition number of the matrix in (6). It is influenced by the choice of (*γ*, *σ*) in the case of RBF LS-SVM. In the following section, we will discuss the algorithm of SMO versions and give the proof of convergence for SD-SMO algorithm.

## 3. SMO and SD-SMO Algorithms for LS-SVM

For solving the LS-SVM problem, the matrix in (6) is usually fully dense and may be too large to be stored. Decomposition methods are designed to handle the difficulties, see Jiao et al. [17]. Unlike other optimization algorithms which update the whole Lagrangian multipliers vector *α* in each iterative process, the decomposition algorithm modifies only a subset of *α* per iteration. We denote the subset as the working set *B*. The SMO algorithm was developed in [10] as a decomposition method to solve the dual problems arising in LS-SVM formulations. In each iteration, SMO algorithm restricts *B* to have only two elements. Because of the problem (4) without the bias term *b*, SMO can be simplified to optimize *B* with only one element at an iteration. By substituting the KKT conditions (5) into the Lagrangian (4), the dual problem is to maximize the following objective function:

(8) $$\begin{array}{c} \max\left(L\left(\alpha \right)\right)=-\frac{1}{2}\sum_{j}\sum_{i}\alpha_{i}\alpha_{j}Q\left(x_{i},x_{j}\right)+\sum_{i}\alpha_{i}y_{i}, \end{array}$$

where *Q*(**x**_*i*_, **x**_*j*_) = *K*(**x**_*i*_, **x**_*j*_) + *σ*_*ij*_/*γ*, and *σ*_*ij*_ = 1 if *i* = *j* and 0 otherwise.

The SMO algorithm for (8) is sketched in the following.

**Algorithm 1**

SMO algorithm for (8) is as follows.

(1) Set *k* = 1 and find *α* ^*k*^ = 0 as the initial feasible solution.
(2) If the stop criterion is satisfied, stop. If not, find a one-element working set *B* = {*i*}⊂{1,…, *N*}. Define *D* ≡ {1,…, *N*}∖*B* and *α*_*B*_^*k*^ and *α*_*D*_^*k*^ to be subvectors of *α*_*k*_ corresponding to *B* and *D*, respectively.
(3) Solve the following subproblem with the variable *α*_*B*_:
  (9) maxαB{−12[αBT(αDk)T][QBBQBDQDBQDD][αBαDk]    +[yBTyDT][αBαDk]},
  where $\left[\begin{array}{cc} Q_{BB} & Q_{BD}\\ Q_{DB} & Q_{DD} \end{array}\right]$ is a permutation of the matrix *Q*.
(4) Set *α*_*B*_^*k*+1^ to be the optimal solution of (9) and *α*_*D*_^*k*+1^ ≡ *α*_*D*_^*k*^. Set *k* ← *k* + 1 and go back to step (2).

In order to find working set *B*, we usually consider whether the KKT conditions is violated or not. The KKT conditions for the dual problem (8) are ∂*L*/∂*α*_*i*_ = 0, which lead to *y*_*i*_ − ∑_*j*_ *α*_*j*_ *Q*(**x**_*i*_, **x**_*j*_) = 0, *i* = 1,2,…, *N*. If we define

(10) $$\begin{array}{c} f_{i}=\frac{\partial L}{\partial \alpha_{i}}=y_{i}-\sum_{j}\alpha_{j}Q\left(x_{i},x_{j}\right), \end{array}$$

then the KKT optimality condition is violated if there exists any index point *i* such that *f*_*i*_ ≠ 0. SMO algorithm for (8) achieves the convergence of optimal process when *f*_*i*_ → 0, for all *i*.

**Figure 1:**
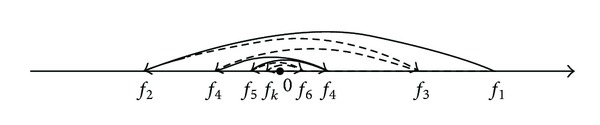
SMO sketch map, where *f*_*k*_ represents the *k*th iteration for *f*_*i*_, for all *i*.

A simple illustration of this is shown in Figure 1. 

Since only one component is updated per iteration, the decomposition method can be quite costly and suffers from slow convergence. For this reason, many researchers improved SMO algorithm. For example, Chen et al. improved SMO algorithm by using the shrinking and caching techniques [18]; Barbero et al. presented a cycle-breaking acceleration of SVM training [16]; and Lin et al. provided three-parameter sequential minimal optimization for support vector machines [19].

As mentioned by Barbero et al. in [16], SMO algorithm is not free of cycle-related problems. For all *i* in working set *B*, if *α*_*i*_ is optimized with step *t* (*t* > 0 or *t* < 0) in a single direction per iteration, the number of cycles in SD-SMO algorithm will be reduced. We now detail SD-SMO formulation in the LS-SVM training process.

Define

(11) $$\begin{array}{c} F_{i}={\left(f_{i}\right)}^{2},\;\forall i. \end{array}$$

Then, the KKT optimality condition is violated if there exists any index point *i* such that *F*_*i*_ ≠ 0.

SD-SMO algorithm works by optimizing only one *α*_*i*_ at each iteration and keeping the others fixed, that is, *α* is adjusted by a sign-invariable step *t*  (*t* > 0  or  *t* < 0) per iteration as follows:

(12) $$\begin{array}{c} \alpha_{i}^{k+1}=\alpha_{i}^{k}+t;\;\alpha_{j}^{k+1}=\alpha_{j}^{k},\;\forall j\neq i. \end{array}$$

The update of *α*_*i*_ causes the change of all the *f*_*j*_ as

(13) $$\begin{array}{c} f_{j}^{k+1}=f_{j}^{k}-\left(\alpha_{i}^{k+1}-\alpha_{i}^{k}\right)Q\left(x_{i},x_{j}\right),\;\forall j \end{array}$$

and; therefore, the function value of *F*_*j*_ will change. At each iteration we need to be sure that the sign of *f*_*j*_^*k*^ is not variable, that is, if *f*_*j*_^*k*^≥ (or ≤) 0, then *f*_*j*_^*k*+1^≥ ( or ≤) 0. As *k* increases, *f*_*j*_^*k*^ → 0^+^ (or 0^−^) with the sign keeping invariable.

**Figure 2:**
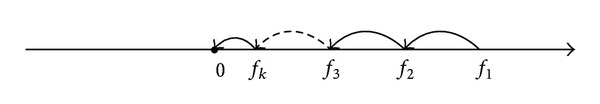
SD-SMO sketch map, where *f*_*k*_ represents the *k*th iteration for *f*_*j*_, for all *j*.

A simple illustration of this is shown in Figure 2.

To derive the optimal step *t* and the termination conditions of iteration, we define *F*_*j*_ as

(14) $$\begin{array}{c} F_{j}\left(t\right)={\left[f\left(\alpha^{\text{new}}\left(t\right)\right)\right]}^{2},\;\;F_{j}\left(0\right)={\left[f\left(\alpha \right)\right]}^{2}. \end{array}$$

Because *f*_*j*_^*k*^ → 0 as *k* → *∞*, *F*_*j*_(*t*) ≤ *F*_*j*_(0). Therefore, let Δ*F*_*j*_ = −(*F*_*j*_(*t*) − *F*_*j*_(0)) and it can be written as

(15) $$\begin{array}{c} \Delta F_{j}=-\left(F_{j}\left(t\right)-F_{j}\left(0\right)\right)=2tf_{j}Q\left(x_{j},x_{j}\right)-t^{2}Q^{2}\left(x_{j},x_{j}\right). \end{array}$$

The optimal step is obtained by maximizing Δ*F*_*j*_ as

(16) $$\begin{array}{c} t^{\text{opt}}=\frac{f_{j}}{Q\left(x_{j},x_{j}\right)}, \end{array}$$

and the optimal step *t* ^opt^ can induce the change of *F*_*j*_ as

(17) $$\begin{array}{c} \Delta F_{j}=\frac{F_{j}}{2Q\left(x_{i},x_{j}\right)}. \end{array}$$

Hence we can choose an index point *j* which has the maximum value of *F*_*j*_/2*Q*(**x**_*j*_, **x**_*j*_) and update *α* by (12) and (16). Suppose *F*(*α*) = (*f*_1_, *f*_2_,…, *f*_*j*_,…, *f*_*N*_) and ||*F*(*α* ^*k*^)||_2_^2^ = ∑_*j*_ *F*_*j*_^*k*^, then {||*F*(*α* ^*k*^)||_2_^2^} is a decreasing sequence. In fact, as *k* → *∞*, ||*F*(*α* ^*k*^)||_2_^2^ → 0. Therefore ||*F*(*α* ^*k*^)||_2_^2^ can be used as a termination criterion for the iterative algorithm as

(18) $$\begin{array}{c} {||F\left(\alpha^{k}\right)||}_{2}^{2}\leq \varepsilon^{2}N, \end{array}$$

where *ε* is a positive constant. The flowchart of SD-SMO algorithm is shown in Algorithm 2.

**Algorithm 2**

SD-SMO algorithm for (8) is as follows.

1. Set *k* = 1 and choose *α* ^*k*^ such that *f*_*j*_ ≥ 0 (or *f*_*j*_ ≤ 0) for all *j* = 1,2,…, *N*.
2. If *α* ^*k*^ satisfies (18), stop. If not, select *p*_1_ = arg⁡max⁡_*j*_(*F*_*j*_/2*Q*(**x**_*j*_, **x**_*j*_))
3. Update *α* ^*k*^ using *t* ^opt^ = *f*_*p*_1__/*Q*(**x**_*p*_1__, **x**_*p*_1__) and (12).
4. While *f*_*p*_1__ ≥ 0 (*f*_*p*_1__ ≤ 0), *k* = *k* + 1, go back to step (2).

One theoretical property of SD-SMO algorithm is presented in the following.

**Theorem 3**

The sequence *α* ^*k*^ generated by SD-SMO algorithm converges to the global optimal solution of (8).

**Proof**

According to the definition of ||*F*(*α* ^*k*^)||_2_^2^ and combining (16) and (17), the following equation holds:

(19) ||F(αk+1)||22−||F(αk)||22=−(topt)2Q(xj,xj)2=−(topt)2(K(xj,xj)+1/γ)2.

The positive-definite kernel function implies *K*(**x**_*j*_, **x**_*j*_) ≥ 0, furthermore ||*α* ^*k*+1^−*α* ^*k*^||_2_^2^ = (*t* ^opt^)^2^, and the following equation is obtained:

(20) $$\begin{array}{c} {||F(\alpha^{k+1})||}_{2}^{2}-{||F(\alpha^{k})||}_{2}^{2}=-\frac{{||\alpha^{k+1}-\alpha^{k}||}_{2}^{2}\left(K\left(x_{j},x_{j}\right)+1/\gamma \right)}{2}. \end{array}$$

Equality (20) yields that {||*F*(*α* ^*k*^)||_2_^2^} is a decreasing sequence. Together with ||*F*(*α* ^*k*^)||_2_^2^ ≥ 0, we have that {||*F*(*α* ^*k*^)||_2_^2^} converges. Applying (20) again, we get that {*α* ^*k*+1^ − *α* ^*k*^} converges to 0 as *k* → *∞*.

Since *F*_*j*_ (∀*j*) is a positive-definite quadratic form, {||*F*(*α*)||_2_^2^} = ∑_*j*_ *F*_*j*_ is a positive-definite quadratic form too. Therefore, the set {*α* | ||*F*(*α*)||_2_^2^ ≤ ||*F*(*α* ^0^)||_2_^2^} is a compact set. {*α* ^*k*^} lies in this set, so it is a bounded sequence. Let $\hat{\alpha }$ be the limit point of any convergent subsequence {*α* ^*k*^}, *k* ∈ Γ. For all *j*, $F_{j}(\hat{\alpha })=\lim_{k\to \infty }\;F_{j}(\alpha^{k})$. According to the definition of *F*(*α* ^*k*^), 0 ≤ *F*_*j*_(*α* ^*k*^) ≤ *F*(*α* ^*k*^). Inequality (18) yields lim⁡_*k*→*∞*_{||*F*(*α* ^*k*^)||_2_^2^} = 0; furthermore, for all *j*, $F_{j}(\hat{\alpha })=\lim_{k\to \infty }F_{j}(\alpha^{k})=0$. While $F_{j}(\hat{\alpha })={\left(f_{j}(\hat{\alpha })\right)}^{2}$, so $f_{1}(\hat{\alpha })=f_{2}(\hat{\alpha }),\ldots$, $=f_{N}(\hat{\alpha })=0$. From the KKT conditions, $\hat{\alpha }$ is the global optimal solution of (8). Since *L*(*α*) is strictly convex, (8) has a unique global solution and we denote it as α*. Assume that {*α* ^*k*^} does not converge to α*. Then, for all *ϵ* > 0, there exists an infinite subset $\tilde{\Gamma }$ such that for all $k\in \tilde{\Gamma }$, ||*α* ^*k*^ − α*|| > *ϵ*. Because {*α* ^*k*^}, for all $k\in \tilde{\Gamma }$ is a compact set, there is a convergent subsequence. Without loss of generality, we assume its limit to be $\hat{\alpha }$. Thus, $||\hat{\alpha }-\alpha^{*}||>\epsilon$. Since $\hat{\alpha }$ is the global optimal solution of (8), this contradicts that $\tilde{\Gamma }$ is the unique global optimal solution. The proof of Theorem is completed.

## 4. Numerical Experiments

 Under the framework Algorithm 2, we conduct experiments to check whether using SD-SMO is really faster than using SMO or not in this section. There have been two techniques for working set selection in SMO-type decomposition methods. The former is first order SMO (FO-SMO) algorithm and the latter is second order SMO (SO-SMO) algorithm for LS-SVM classifiers [20]; that is, the former uses first order information to achieve fast convergence and the latter uses second order information. Two groups of experiment have been done in order to compare SD-SMO with the above two algorithms. All methods are implemented in MATLAB and executed on a personal computer with Intel(R) Core(TM) i3 2.53 GHz processors, 2.00-GB memory, and Windows 7 operation systems. For all algorithms, the optimization process is terminated when the maximal violation of the KKT conditions is within *ε* = 0.001. For simplicity, we consider only Gaussian kernel *k*(**x**, **x**_*k*_) = exp⁡{−||**x**−**x**_*k*_||_2_^2^/2*σ* ^2^} to construct LS-SVM.

### 4.1. The Comparison of SD-SMO with First Order SMO

 In this section, we compare SD-SMO with first order SMO on four benchmark datasets for evaluating the performance of the proposed method. We compare the two methods in terms of computational cost, which is measured by the number of iteration. The examples introduced by Keerthi and Shevade [10] are used. Datasets used for this purpose are Banana, Image, Waveform, and Splice. For each dataset, the value of *σ* ^2^ is determined by the five-fold cross validation on a small random subset.

In the first experiment, we vary *γ* over a small range because the extremely small and large *γ* values are usually of little interest. We try the following nine *γ* values: 2^*i*^, *i* = −4, −3,…, 3,4. In Table 1, the computational costs associated with the four datasets as functions of *γ* are given when the optimization process is terminated.

 As a basis for the comparisons, Table 1 shows the computational costs of first order SMO and SD-SMO algorithms at different values of parameter *γ*. For first order SMO algorithm, the computational cost increases with the increase of *γ*. While for SD-SMO algorithm, it is not so. For instance, see the computational cost of SD-SMO for the Banana and Waveform datasets. From Table 1, we can see that the number of iterations of SD-SMO algorithm is much smaller than that of first order SMO one, especially for Image dataset.

In order to further show the performance of SD-SMO algorithm, Tables 2 and 3 are given. The tables report the training time and the generalization performance of first order SMO and SD-SMO algorithms for four benchmark datasets. The generalization performance is illustrated by the classification accuracy of an independent test set for each dataset.

From Tables 2 and 3, we can see that the generalization capabilities of both methods are comparable, but the training time of SD-SMO algorithm is shorter than first order SMO algorithm. For instance, in the case of Image dataset, the training time for first order SMO algorithm with the best generalization performance is 41.6108 s. It represents the equivalent of ten times the cost of SD-SMO algorithm. The classification accuracy for Image dataset with SD-SMO algorithm is 0.963, and it is almost equal to the one with first order SMO algorithm. In consequence, the efficacy and feasibility of the proposed SD-SMO algorithm is superior to that of first order SMO one for LS-SVMs.

### 4.2. The Comparison of SD-SMO with Second Order SMO

 To further explore the performance of the proposed method, we compare SD-SMO with second order SMO by a second set of experiments on the datasets Titanic, Heart, Breast Cancer, Thyroid, and Pima (available in [21]). We use the datasets provided in [21] to certify the good generalization properties of the proposed method. In Table 4, the number of iterations and execution times per experiment is reported. The misclassification rates are also reported in Table 4.

It can be seen that for these datasets it is better to use SD-SMO in Cancer, Pima, and Titanic. The results in Table 4 shows that the biggest improvement with SD-SMO happens for Titanic. Therefore, this is further evidence on the previous observation that for large-scale problems SD-SMO outperforms second order SMO.

The final set of experiments aims to ascertaining how well the SMO algorithm scales for large-scale datasets when it uses the different working set selections. In order to test this, we use the datasets a8a and covtype.binary, available with several increasing numbers of patterns in [22].

**Figure 3:**
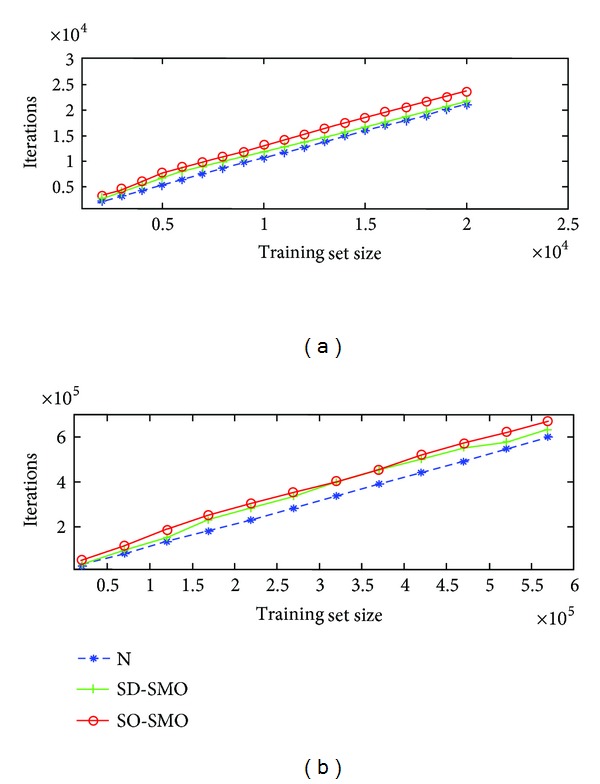
Variation of the number of iterations with training set size for a8a (a) and covtype (b).

In Figure 3, we plot the results for a8a with *C* = 2, *σ* ^2^ = 10 and covtype.binary with *C* = 10, *σ* ^2^ = 10, respectively. As it can be seen, the number of iterations scales linearly with the training set size. Note that SD-SMO needs less iterations to convergence, as expected. And the reduction is greater for covtype.binary because of its larger value of *C*. In any case, the scaling is linear in both cases.

## 5. Conclusion

 In this paper, a new algorithm, that is, SD-SMO, is proposed. It can be used to select working set for LS-SVM classifier training, and its asymptotic convergence is proved theoretically. Based on SMO formulation, the path of one-side convergence is used effectively in our method. The number of iterations and kernel operations in SD-SMO algorithm is less than that of the traditional SMO algorithm, so the new algorithm provides faster convergence speed. Simulation experiments have been carried out on four benchmark datasets. The empirical comparisons demonstrate that SD-SMO algorithm is much more efficient in terms of computational time than first order and second order SMO, and at the same time there are no large differences in terms of accuracy.

## Figures and Tables

**Table 1:** Computational costs for first order SMO (FO-SMO) and SD-SMO algorithms.

log⁡_2_^*γ*^	Banana		Image		Waveform		Splice	
	*σ* ^2^ = 1.8221		*σ* ^2^ = 2.7183		*σ* ^2^ = 24.5325		*σ* ^2^ = 29.9612	
	FO-SMO	SD-SMO	FO-SMO	SD-SMO	FO-SMO	SD-SMO	FO-SMO	SD-SMO
−4	0.4460	0.3548	0.4838	0.1104	0.5375	0.2234	0.4375	0.3166
−3	0.5023	0.3542	0.5150	0.1191	0.5854	0.2499	0.4683	0.3152
−2	0.6379	0.3381	0.5844	0.1217	0.6109	0.2343	0.5066	0.3029
−1	0.8733	0.2632	0.7413	0.1248	0.6682	0.2245	0.6060	0.2662
0	1.3545	0.2231	0.9816	0.1283	0.7440	0.1879	0.7738	0.2105
1	2.3782	0.1607	1.4816	0.1326	0.8512	0.1672	1.3078	0.1775
2	2.4793	0.0679	1.8371	0.2927	0.9569	0.1490	1.3537	0.1675
3	2.6521	0.0486	2.3751	0.2136	1.0829	0.1369	1.7175	0.1481
4	2.8906	0.0231	2.9305	0.2205	1.2195	0.1344	2.1520	0.1402

Note: each unit corresponds to 10^4^ iterations.

**Table 2:** Training time (in seconds) and classification accuracy in parentheses for first order SMO (FO-SMO) and SD-SMO algorithms.

log⁡_2_^*γ*^	Banana		Image	
	*σ* ^2^ = 1.8221		*σ* ^2^ = 2.7183	
	FO-SMO	SD-SMO	FO-SMO	SD-SMO
−4	43.6589 (0.8675)	**35.947 (0.895) **	7.90140 (0.9012)	2.47260 (0.9214)
−3	47.3385 (0.8753)	35.3045 (0.8712)	8.41620 (0.9156)	2.50380 (0.9324)
−2	59.8110 (0.8832)	34.3882 (0.8653)	9.76570 (0.9223)	2.59740 (0.9348)
−1	88.6335 (0.8889)	28.9070 (0.8377)	11.7874 (0.9382)	2.57400 (0.9358)
0	129.505 (0.8877)	22.5036 (0.8667)	15.3895 (0.9430)	2.58180 (0.9410)
1	220.437 (0.8900)	16.1617 (0.8502)	23.4157 (0.9521)	2.60520 (0.9511)
2	229.891 (0.8943)	8.42400 (0.7853)	31.1026 (0.9588)	3.93120 (0.9602)
3	238.068 (0.8977)	3.47140 (0.7032)	**41.611 (0.967) **	**4.2979 (0.963) **
4	**259.36 (0.898) **	2.02800 (0.6126)	50.6560 (0.9616)	4.50900 (0.9578)

**Table 3:** Training time (in seconds) and classification accuracy in parentheses for first order SMO (FO-SMO) and SD-SMO algorithms.

log⁡_2_^*γ*^	Waveform		Splice	
	*σ* ^2^ = 24.5325		*σ* ^2^ = 29.9612	
	FO-SMO	SD-SMO	FO-SMO	SD-SMO
−4	43.4541 (0.9094)	35.4434 (0.8404)	31.9303 (0.8649)	44.4478 (0.6507)
−3	46.5039 (0.9108)	36.1884 (0.8918)	33.2688 (0.8736)	44.0110 (0.7061)
−2	48.8049 (0.9114)	**37.635 (0.908) **	36.0175 (0.8910)	41.9830 (0.8944)
−1	**52.907 (0.912) **	35.3499 (0.8948)	43.6085 (0.8963)	**37.730 (0.911) **
0	58.9295 (0.9096)	29.9522 (0.8974)	55.2503 (0.9037)	33.4865 (0.8866)
1	67.2830 (0.9071)	26.6060 (0.8955)	**72.543 (0.911) **	26.1801 (0.8826)
2	79.3185 (0.9068)	24.5008 (0.8859)	94.8392 (0.9060)	23.3596 (0.8769)
3	86.3930 (0.9004)	22.9251 (0.8876)	121.219 (0.9054)	21.7434 (0.8750)
4	95.7465 (0.9100)	22.4251 (0.8860)	153.243 (0.9032)	21.0508 (0.8746)

**Table 4:** Number of iterations (in thousands), execution times (in seconds), and average misclassification rates for second order SMO (SO-SMO) and SD-SMO algorithms.

Dataset	Iterations		Executiontimes		Misclassification rate	
	SO-SMO	SD-SMO	SO-SMO	SD-SMO	SO-SMO	SD-SMO
Titanic	277.1512	59.7346	1129.2009	80.9348	23.5723	23.5612
Heart	5.8993	2.2315	10.3623	4.4652	16.1117	17.1092
Cancer	10.1908	4.1127	21.6765	9.0972	27.6643	27.8764
Thyroid	30.1537	17.7325	77.3341	52.5521	5.5123	5.6725
Pima	60.6751	30.7366	104.9616	69.8546	25.0155	25.7761
